# Two-Year Visual Outcomes of Evolution Implantable Collamer Lens and Small Incision Lenticule Extraction for the Correction of Low Myopia

**DOI:** 10.3389/fmed.2022.780000

**Published:** 2022-04-13

**Authors:** Mengjun Fu, Meiyan Li, Yiyong Xian, Zhiqiang Yu, Haorun Zhang, Joanne Choi, Lingling Niu, Xiaoying Wang, Xingtao Zhou

**Affiliations:** ^1^Weifang Eye Hospital, Weifang, China; ^2^Eye Institute and Department of Ophthalmology, Eye & ENT Hospital, Fudan University, Shanghai, China; ^3^NHC Key Laboratory of Myopia, Fudan University, Shanghai, China; ^4^Key Laboratory of Myopia, Chinese Academy of Medical Sciences, Shanghai, China; ^5^Shanghai Research Center of Ophthalmology and Optometry, Shanghai, China; ^6^Wayne State University School of Medicine, Detroit, MI, United States

**Keywords:** smile, EVO-ICL, Low myopia, visual quality, aberration

## Abstract

**Purpose:**

To investigate the 2-year visual quality of Evolution Implantable Collamer Lens (EVO-ICL) and small incision lenticule extraction (SMILE) for the correction of low myopia.

**Methods:**

In this prospective study, we included 25 eyes of 25 patients (7 men) who underwent EVO-ICL and 36 eyes of 36 patients (16 men) who underwent SMILE between January 2018 and December 2018. Subjective and objective visual outcomes were compared between ICL and SMILE. All patients were followed for 2 years.

**Results:**

At the postoperative 2-year visit, the percentage of patients with uncorrected distance visual acuity (UDVA) greater than or equal to preoperative corrected distance visual acuity (CDVA) was comparable in the ICL group (80%, 20/25) and SMILE group (88.89%, 32/36). Spherical equivalent (SE) was within ± 0.50 D in 96% (24/25) of the ICL group and 94.44% (34/36) of the SMILE group. No eyes lost more than 2 lines of CDVA. Postoperative high-order aberrations (HOAs) were significantly increased in the ICL group (*p* < 0.01) and in the SMILE group (*p* < 0.01). The most common visual complaint was halo after ICL and starburst after SMILE. There was no correlation between HOAs and visual complaints (*p* > 0.05).

**Conclusion:**

Evolution Implantable Collamer Lens provides comparable safety, efficacy, long-term visual stability, and high patient satisfaction when compared to SMILE in correcting low myopia. EVO-ICL could be a favorable alternative for low myopia.

**Key messages What was known?:**

• Visual outcomes of Evolution Implantable Collamer Lens (EVO-ICL) versus small incision lenticule extraction (SMILE) for correction of mild myopia remain unclear.

**What this paper adds?:**

• Evolution Implantable Collamer Lens (EVO-ICL) provides comparable safety, efficacy, long-term visual stability, and high patient satisfaction when compared to small incision lenticule extraction (SMILE) in correcting low myopia.
• The most common visual complaint was halo after ICL and starburst after SMILE.

## Introduction

In the past two decades, innovations in refractive surgery have rapidly developed. Small incision lenticule extraction (SMILE) and Evolution Implantable Collamer Lens (EVO-ICL) are two such examples. The advantages of SMILE come from the flapless design, which results in a less degree of postoperative dry eye and offers biomechanical stability. SMILE has been shown to have good safety, efficacy, and predictability, but only patients with corneas of an adequate thickness and without irregularities are eligible for this surgery. Unlike SMILE, the EVO-ICL does not remove corneal tissue and instead corrects the refractive error by implanting a collamer lens in phakic eyes. It provides a wider range of myopic correction (0 to –18.00 D) and is minimally invasive with a short recovery time. Due to this, EVO-ICL has quickly gained mainstream recognition as the surgery of choice for the correction of high myopia (1–3). However, presently there are only a few studies that address the usage of EVO-ICL for correction of mild to moderate myopia. These studies compared EVO-ICL to laser-assisted *in situ* keratomileusis (LASIK) and found that ICL was safer, more efficacious, predictable, and demonstrated more stability (4, 5). In addition, ICL introduced less high-order aberrations (HOAs) and had better contrast sensitivity than LASIK (5).

Current literature mainly focuses on comparing the visual outcomes of ICL and SMILE. To the best of our knowledge, there have been no studies comparing EVO-ICL and SMILE to correct mild myopia. This study reports the 2-year visual outcomes of EVO-ICL versus SMILE for low myopia correction.

## Materials and Methods

In this prospective study, we included 25 eyes of 25 patients (7 men) with a mean spherical equivalent (SE) within –3.04 ± 0.59 diopters (D) who underwent EVO-ICL, and 36 eyes of 36 patients (16 men) with a mean SE within –2.95 ± 0.73 D who underwent SMILE between January 2018 and December 2018 at the Fudan University Eye and ENT Hospital (Shanghai, China). All patients were enrolled in the monocular group. If both eyes met the inclusion criteria, the right eye was selected (Table 1).

**TABLE 1:** Demographic data and preoperative characteristics.

	ICL group (*n* = 25 eyes)		SMILE group (*n* = 36 eyes)		*P*-values	
	Mean ± SD	Range	Mean ± SD	Range	*t*	*P*
Age(years)	28.8 ± 4.7	19, 36	29.4 ± 5.9	21, 40	–0.19	0.85
Gender(male/female)	7/18			16/20	0.17	0.19
Sphere(D)	–2.69 ± 0.52	–3.00, –1.00	–2.58 ± 0.59	–3.00, –1.00	0.73	0.47
Cylinder(D)	0.70 ± 0.54	0.00, 1.50	0.73 ± 0.44	0.00, 1.50	0.23	0.82
SE(D)	–3.04 ± 0.59	–3.75, –1.50	–2.95 ± 0.73	–3.75, –1.13	0.54	0.60
IOP(mmHg)	14.42 ± 2.36	9.70, 19.10	15.34 ± 2.93	9.30, 20.90	1.30	0.20
AL(mm)	24.85 ± 0.80	23.00, 26.85	25.11 ± 0.64	23.94, 26.29	1.39	0.17

*ICL, Implantable Collamer Lens; SMILE, small incision lenticule extraction; SE, spherical equivalent; D, diopters; IOP, intraocular pressure; AL, axial length.*

### Inclusion and Exclusion Criteria

For patients with low myopia, we recommend laser refractive surgery as the first choice if the patient could have laser refractive surgery. EVO-ICL was recommended for patients with low myopia who desired myopic correction but were not suitable candidates for corneal refractive surgeries due to insufficient corneal thickness (less than 480 μm) or high risk of postoperative keratectasia.

Inclusion criteria were as follows: (1) age 20–40 years old; (2) spherical –0.50 to –3.00 D, cylinder ≤ 1.50 D, corrected distance visual acuity (CDVA) ≥ 20/20; (3) stable refractive error (annual change ≤ 0.50 D in the past 2 years); (4) the anterior chamber depth (ACD) ≥ 2.8 mm and endothelial cell density (ECD) ≥ 2,000/mm^2^ in the patients who underwent EVO-ICL surgery; the residual corneal stromal bed thickness ≥ 280 μm in the patients who underwent SMILE surgery; and (5) contact lens use was discontinued for 1 week for soft contact lenses and 2 or more weeks for Rigid Gas Permeable contact lenses.

Exclusion criteria were as follows: (1) suspicious keratoconus; (2) a history of eye trauma and prior eye surgery; and (3) other eye diseases and systemic diseases affecting the eyes.

### Main Refractive and Biometric Measures

Routine ophthalmic examinations were performed preoperatively and postoperatively that include (1) slit lamp and fundus examination; (2) uncorrected distance visual acuity (UDVA) and CDVA; (3) measurement of the axis (Humphrey IOL Master, Carl Zeiss Meditec, Germany) and intraocular pressure (IOP); (4) corneal topography (Pentacam HR, Type 70900; Oculus Optikgerate GmbH, Wetzlar, Germany); (5) Wavefront Supported Custom Ablation (WASCA) Wavefront Analyzer: deviational data were analyzed at 6.0-mm scale using Zernike polynomials under scotopic conditions without pharmacological pupillary dilation. The root means square (RMS) values of total HOAs, spherical aberration, coma aberration (i.e., vertical and horizontal coma), and trefoil aberration (i.e., vertical and oblique trefoil) were calculated (Carl Zeiss Meditec, Germany); (6) principal optometry and computer optometry with pupil dilation. For patients who received EVO-ICL implantation, the horizontal diameter of the cornea, the diameter of the ciliary groove [Ultrasound Biomicroscope (UBM), Quantel Medical, France], and the minimum ACD were examined preoperatively, and the vault was examined postoperatively (Pentacam). ECD was measured preoperatively and postoperatively (SP-2000P, Topcon Corporation, Japan).

### Subjective Visual Quality

A questionnaire was used to ask patients about their subjective visual quality. The questionnaire contained 6 common visual symptoms, i.e., glare, halo, starburst, hazy vision, blurred vision, and fluctuation vision. Questions about the first five symptoms also provided example images to reduce the likelihood of question misinterpretation. In addition, the questionnaire asked patients about their overall satisfaction, improvement in visual acuity, and whether they would recommend EVO-ICL or SMILE to patients with similar conditions.

### Surgical Techniques

#### Evolution Implantable Collamer Lens

The ICL power was calculated using the STAAR Surgical Online Calculator (STAAR Surgical, Nidau, Switzerland). In this study, the degree of the sphere was sufficiently corrected. When the cylinder was ≥ 1.00 D, a Toric ICL (TICL) was selected. When the cylindrical was 0.50–1.00 D, the patient was tried on glasses. If correcting cylinder did not improve a patient’s CDVA, an ICL was selected; if it did improve CDVA, TICL was selected. ICL was selected when the cylindrical was less than 0.50 D. The size of the ICL was selected based on a patient’s white-to-white (WTW) horizontal diameter, ciliary sulcus horizontal diameter, and ACD.

The one-step technique was applied, in which only one 3.0-mm corneal incision was made and a viscoelastic agent was injected only once between the ICL lens and the cornea after ICL lens injection. This technique for ICL implantation has previously been described by Wei et al. (6).

#### Small Incision Lenticule Extraction Surgery

Small incision lenticule extraction was performed in expert mode using a 500 kHz VisuMax femtosecond laser system (Carl Zeiss Meditec AG, Jena, Germany). All eyes were treated with S-size cone, repetition frequency 500 kHz, pulse energy 130 nJ, corneal cap thickness 120 μm, optical region diameter 6.5–6.8 mm, and base thickness 10 μm. The cut was set at 90° (12:00 clock) and the width was 2.0 mm. A spot distance of 2.5 μm was used for the lenticule cut and cap cut, and a spot distance of 2.0 μm was used for the lenticule side-cut and cap side-cut. The SMILE surgical procedure had previously been described by Li et al. (7).

### Postoperative Medication and Nursing

The following eye drops were used in the EVO-ICL group: 0.5% levofloxacin (Cravit; Santen, Osaka, Japan) four times daily for 7 days; 1.0% prednisolone acetate (Pred Forte; Allergan, Irvine, CA, United States) four times a day for 4 days; Pranoprofen (Senju, Osaka, Japan) was used four times a day for 14 days; and artificial tears (Hyalein, 0.1% hyaluronic acid, Santen) four times daily for 1 month.

The following eye drops were used in the SMILE group: 0.5% levofloxacin (Cravit; Santen, Osaka, Japan) four times daily for 7 days; 0.1% fluorometholone (Fluorometholone; Santen, Osaka, Japan) eight times daily and tapered to one time daily for over 24 days; and artificial tears (Hyalein, 0.1% hyaluronic acid, Santen) four times daily for 3 months.

The length of follow-up was 2 years.

### Statistical Analysis

Statistical analysis was performed using R version 3.6.2 (R Project for Statistical Computing). Continuous variables were expressed as mean ± standard deviation (SD), whereas categorical variables were represented as frequency and percent. Wilcoxon was used to compare age and gender between groups. Shapiro–Wilk test was executed to examine the data for normal distribution. For normally distributed variables, independent *t*-tests were performed, whereas the Wilcoxon test was applied for variables that were not normally distributed. The Chi-square test was used to assess the statistical significance of differences in percentages. Logistic regression analysis was used to analyze the correlation between HOAs and Vision Distributions. Values of *p* less than 0.05 were considered to indicate statistically significant differences.

## Results

All surgeries were uneventful and without any complications, such as infection (Table 2).

**TABLE 2:** Postoperative parameters of the two groups.

	ICL group (*n* = 25 eyes)		SMILE group (*n* = 36 eyes)		*P*-values	
	Mean ± SD	Range	Mean ± SD	Range	*t*	*P*
UDVA (LogMAR)	–0.01 ± 0.05	–0.10, 0.05	–0.08 ± 0.07	–0.20, 0.05	–4.23	<0.01
CDVA (LogMAR)	–0.06 ± 0.05	–0.10, 0	–0.10 ± 0.06	–0.20, 0.05	–3.41	<0.01
Efficacy indices	1.02 ± 0.10	0.90, 1.20	1.17 ± 0.17	0.90, 1.50	–3.48	<0.01
Safety indices	1.11 ± 0.10	1.00, 1.20	1.23 ± 0.15	0.90, 1.50	–3.30	<0.01
Sphere error(D)	0.15 ± 0.22	–0.50, 0.25	–0.08 ± 0.16	–0.50, 0	1.32	0.20
cylinder error(D)	0.14 ± 0.23	0.00, 0.75	0.07 ± 0.13	0.00, 0.50	–1.40	0.17
Residual SE(D)	–0.22 ± 0.24	–0.63, 0.13	–0.12 ± 0.19	–0.50, 0	1.82	0.09
IOP(mmHg)	14.46 ± 2.20	9.90, 19.40	12.77 ± 2.33	9.20, 20.30	–2.85	<0.01
AL (mm)	24.82 ± 0.83	22.89, 26.94	25.05 ± 0.64	23.88, 26.22	1.24	0.22

*ICL, Implantable Collamer Lens; SMILE, small incision lenticule extraction; UDVA, uncorrected distance visual acuity; CDVA, corrected distance visual acuity; D, diopters; IOP, intraocular pressure; AL, axial length.*

### Efficacy

**FIGURE 1:**
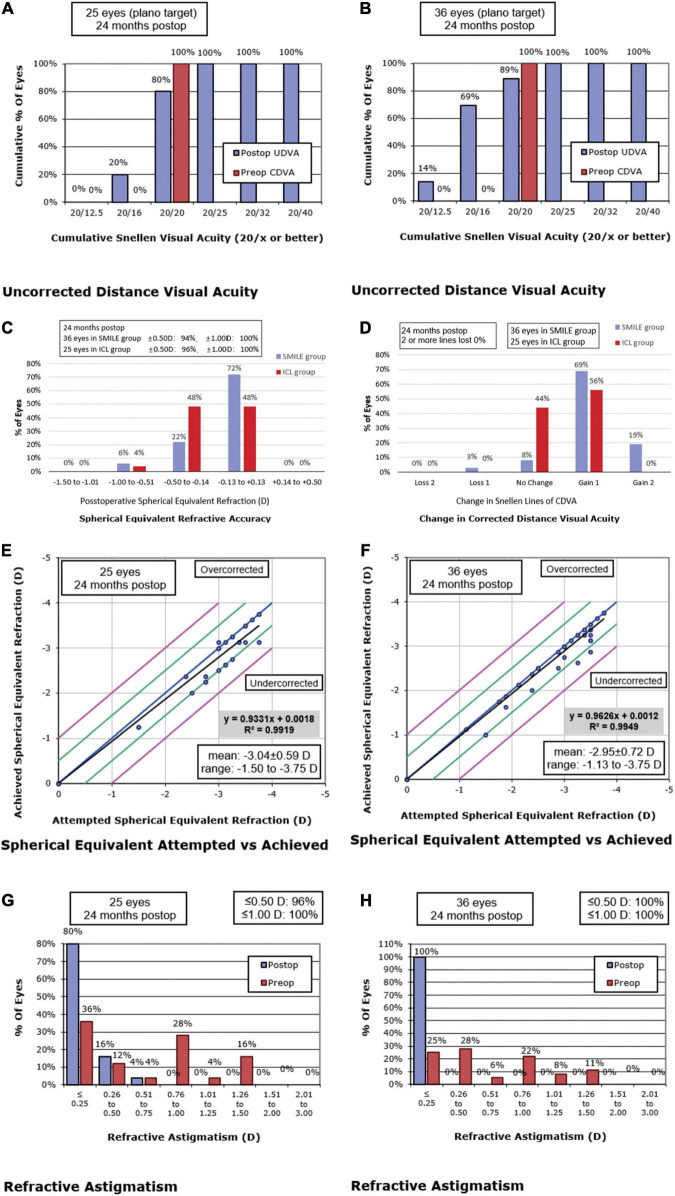
Postoperative 2-year refractive outcomes after Implantable Collamer Lens (ICL) **(A,C,E,G)** and small incision lenticule extraction (SMILE) **(B, D, F, H)** for myopia. Cumulative uncorrected distance visual acuity (UDVA) after ICL **(A)** and SMILE **(B)**. **(C)** Spherical equivalent refraction after ICL and SMILE. **(D)** Changes in corrected distance visual acuity (CDVA) after ICL and SMILE. Attempted versus achieved spherical equivalent refraction after ICL **(E)** and SMILE **(F)**. Postoperative refractive astigmatism after ICL **(G)** and SMILE **(H)**. postop: postoperative; preop: preoperative.

The postoperative efficacy indexes (postoperative UDVA/preoperative CDVA) of the ICL group and SMILE group were 1.02 ± 0.10 and 1.17 ± 0.17, respectively (*p* < 0.01). LogMAR UDVA was ≤ 0 in 80% (20/25) of ICL eyes and 88.89% (32/36) of SMILE eyes. LogMAR UDVA of all operative eyes in both groups was ≤ 0.10. Postoperative UDVA was equal to or better than preoperative CDVA in 80% (20/25) of ICL eyes and 88.89% (32/36) of SMILE eyes (Figure 1).

### Safety

The safety indexes (postoperative CDVA/preoperative CDVA) of the ICL group and SMILE group were 1.11 ± 0.10 and 1.23 ± 0.15, respectively (*p* < 0.01). CDVA was improved by one line in 56% (14/25) of ICL eyes and 69.44% (25/36) of SMILE eyes. In both groups, no eyes lost two or more lines of CDVA (Figure 1). In the ICL group, the preoperative ECD was 2559.64 ± 252.29 cells/mm^2^, and the ECD at 2 years postoperatively was 2595.84 ± 242.42 cells/mm^2^. There was no statistically significant difference in ECD before and after surgery (*p* = 0.40). During the 2-year follow-up, the average vault in the ICL group was 470.80 ± 179.84 μm, and 88% (22/25) of ICL eyes maintained the ideal vault from 250 to 750 μm. No cataract was observed.

### Predictability

The postoperative SE was within ± 0.25 D in 60% (15/25) of ICL eyes and 80.56% (29/36) of SMILE eyes. SE was within ± 0.50 D in 96% (24/25) of ICL eyes and 94.44% (34/36) of SMILE eyes. The SE of all eyes in both groups was within ± 1.0 D (Figure 1). Residual astigmatism was within 0.25 D in 80% (20/25) of ICL eyes and 100% (36/36) of SMILE eyes. Residual astigmatism was within 0.75 D in all eyes (Figure 1). There were no significant differences in the residual sphere, residual cylinder, and residual SE between the ICL group and the SMILE group (all *p* > 0.05).

### Ocular Wavefront Aberration (In a 6.0-mm Pupil)

There was a statistically significant increase in postoperative HOAs in both the ICL group and SMILE group (*p* < 0.01). There were no significant differences in spherical aberration, coma aberration, and trefoil aberration between preoperative and postoperative measurements in the ICL group (all *p* > 0.05). Postoperative spherical aberration and trefoil aberration in the SMILE group showed no significant difference when compared to corresponding values before surgery (all *p* > 0.05), but HOAs and coma (especially vertical coma) in the SMILE group were increased after surgery (*p* < 0.01). The change in coma (postoperative values minus preoperative values) was smaller in the ICL group when compared to the SMILE group (*p* = 0.02 for coma, *p* = 0.04 for vertical coma; Table 3).

**TABLE 3:** Induced changes in aberrations before and after surgery.

	Preoperative		Postoperative		ΔICL	ΔSMILE	*t*	*P*
	ICL	SMILE	ICL	SMILE				
HOAs	0.36 ± 0.15	0.34 ± 0.14	0.66 ± 0.46	0.82 ± 0.53	0.30 ± 0.44	0.49 ± 0.56	1.30	0.31
Z 4,0	0.13 ± 0.07	0.09 ± 0.12	0.13 ± 0.16	0.12 ± 0.10	0.00 ± 0.16	0.03 ± 0.15	0.14	0.89
Z 3,–3	–0.07 ± 0.13	–0.07 ± 0.14	–0.06 ± 0.13	–0.07 ± 0.14	0.00 ± 0.18	0.00 ± 0.14	–0.65	0.52
Z 3,-1	0.03 ± 0.21	0.07 ± 0.14	–0.00 ± 0.16	–0.10 ± 0.18	–0.03 ± 0.11	–0.17 ± 0.18	–4.27	0.00
Z 3, 1	0.01 ± 0.16	0.006 ± 0.10	–0.01 ± 0.22	0.08 ± 0.25	–0.02 ± 0.19	0.07 ± 0.27	1.24	0.23
Z 3, 3	–0.00 ± 0.13	–0.01 ± 0.11	–0.05 ± 0.18	–0.01 ± 0.13	–0.05 ± 0.16	0.00 ± 0.12	1.12	0.26

*ICL, Implantable Collamer Lens; SMILE, small incision lenticule extraction; HOAs, high-order aberrations. Δ Difference between postoperative to preoperative aberrations.*

### Correlation Between Postoperative Visual Disturbances and Objective Indicators

**FIGURE 2:**
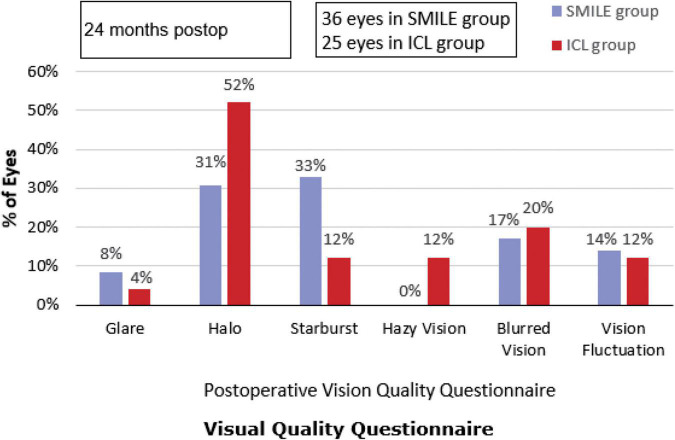
Subjective visual quality after Implantable Collamer Lens (ICL) and small incision lenticule extraction (SMILE). postop: postoperative.

At the end of the 2-year follow-up, 96% (24/25) of patients with ICL and 91.67% (33/36) of patients with SMILE reported significant improvement in their visual quality, and 92% (23/25) of patients with ICL and 91.67% (33/36) of patients with SMILE were very satisfied with their visual outcomes. The most commonly reported visual disturbances in the ICL group were halo, blurred vision, and hazy vision. The most commonly reported visual disturbances in the SMILE group were starburst, halo, and blurred vision. The incidence of postoperative halo in the ICL group was significantly higher than in the SMILE group, while the incidence of postoperative starburst was significantly higher in the SMILE group than in the ICL group (Figure 2). There were no significant associations between HOAs and reported subjective visual disturbances (all *p* > 0.05). Most patients had only occasional and mild visual complaints after surgery, and these symptoms did not affect their quality of life. In total, 96% (24/25) of the ICL group and 97.22% (35/36) of the SMILE group were willing to recommend the operation to myopic patients with similar conditions, while the rest chose “not sure whether to recommend the operation” due to cost considerations.

## Discussion

The efficacy of SMILE surgery in correcting low myopia and myopic astigmatism had been widely recognized (8–12). In recent years, few studies have also reported that EVO-ICL demonstrated good efficacy and safety in correcting low to moderate myopia (13, 14). This study first investigated and compared the long-term visual outcomes between EVO-ICL surgery and SMILE surgery for low myopia.

In the 2-year follow-up period, both ICL and SMILE surgeries showed good safety and efficacy, which is consistent with the results of previous studies (2, 3, 15–17). In this study, EVO-ICL achieved comparable outcomes for low myopia correction. This was closely related to surgical planning and accurate EVO-ICL power calculation. In the selection of ICL for the correction of low myopia, the spherical error was sufficiently corrected. When the cylinder was ≥ 1.00 D, TICL was selected. When the cylindrical was between 0.50 and 1.00 D, the patient was tried on glasses. If the cylindrical error was not corrected and the patient’s CDVA was not affected, ICL was selected, otherwise, TICL was selected. ICL was used when the cylindrical error was less than 0.50 D. The results of this study showed that SE was within ± 0.5 D in 96% of the ICL eyes, and within ± 1.0 D in all the eyes, indicating that ICL has good predictability in correcting low myopia.

High-order aberrations were increased after both ICL and SMILE surgeries. However, when compared to SMILE, ICL induced fewer HOAs, especially coma, which is consistent with previous studies (1, 18–20). Aruma et al. (1) and Wei et al. (6) previously reported that when compared with SMILE surgery, ICL introduced less total HOAs, coma, and spherical aberrations [Aruma et al. (1): in moderate myopic patients; Wei et al. (6): in high myopic patients]. This makes sense given that ICL is an intraocular refractive surgery that does not require ablation of corneal tissue and does not cause flattening of the anterior surface curvature of the cornea.

In this study, although the overall satisfaction after ICL and SMILE was relatively high, there were still some patients with visual complaints after refractive surgery. The incidence of halo after ICL was significantly higher than after SMILE. This is likely related to the central hole in EVO-ICL. Aruma et al. (1) and Wei et al. (6) reported that the most common visual disturbance after ICL for high myopic correction was the halo. Eppig et al. (21) suggested that the central hole in the ICL might be form an additional optical interface that might cause light transmissibility, especially in hyperopic or moderate-to-low-sighted eyes. However, Shimizu et al. (22) reported that the central hole of the EVO-ICL did not produce any more halo than conventional ICL. In this study, the most common visual complaint after SMILE for low myopia correction was starburst, which differs from prior studies on SMILE for high myopia correction. Aruma et al. (1) and Wei et al. (6) reported that the most common complaint after SMILE was blurred vision. This may be explained by the fact that more ablation of corneal tissue is necessary to correct moderate and high myopia, causing the corneal curvature to become flattered after surgery and reduce tear film stability. In this study, although the patients had different visual complaints, the visual quality problems did not affect their quality of life.

There are several limitations to this study. In this study, only patients with sphere ≤ –3.00 D and cylinder ≤ 1.5 D were included. Therefore, the results of this study cannot be extrapolated to patients with spherical > –3.00 D or astigmatism > 1.5 D. The power of ICL ranges from –0.50 to –18.00 D and is available in 0.25 D increments for ICLs between –0.50 to –3.00 D and in 0.50 D increments for lenses between –3.00 to –18.00 D. In contrast, TICL is only available from –3.0 to –18.0 D in 0.5 D increments. In this study, TICLs were only selected when the cylinder was ≥ 1.0 D and when the degree of astigmatism was between 0.50 and 1.0 D. In addition, the sample size was limited. For patients with low myopia, if laser refractive surgery was available, we would recommend laser refractive surgery first. ICL was only recommended for patients with thin and irregular corneas and a strong desire to be glasses-free, so the number of patients in this cohort was relatively small. Additionally, a contrast sensitivity test was not performed and future studies on contrast sensitivity are needed.

In conclusion, EVO-ICL and SMILE provided good safety, efficacy, long-term stability, and high patient satisfaction in correcting low myopia. EVO-ICL could be a favorable alternative for low myopia correction.

## Data Availability Statement

The original contributions presented in the study are included in the article/supplementary material, further inquiries can be directed to the corresponding authors.

## Ethics Statement

The studies involving human participants were reviewed and approved by the Ethics Committee of Fudan University’s EENT Hospital Review Board. The patients/participants provided their written informed consent to participate in this study.

## Author Contributions

MF, ML, and YX conducted the study. MF, ML, and JC wrote the manuscript. YX, LN, and HZ contributed to the data collection. XW and XZ revised the manuscript and designed the study. All authors contributed to the article and approved the submitted version.

## Conflict of Interest

The authors declare that the research was conducted in the absence of any commercial or financial relationships that could be construed as a potential conflict of interest.

## Publisher’s Note

All claims expressed in this article are solely those of the authors and do not necessarily represent those of their affiliated organizations, or those of the publisher, the editors and the reviewers. Any product that may be evaluated in this article, or claim that may be made by its manufacturer, is not guaranteed or endorsed by the publisher.
